# Reviewer acknowledgement

**DOI:** 10.1186/1750-1326-8-11

**Published:** 2013-04-02

**Authors:** Guojun Bu, Huaxi Xu

**Affiliations:** 1 Department of Neuroscience, Mayo Clinic, Jacksonville, Florida, USA; 2 Sanford-Burnham Medical Research Institute, La Jolla, California, USA

## Abstract

**Contributing reviewers:**

The editors of Molecular Neurodegeneration would like to thank all the reviewers who have contributed to the journal in Volume 7 (2012).

## 

Samer Abdul-Hay

United States of America

Hiroyoshi Ariga

Japan

Marco Atzori

United States of America

Corinne Augelli-Szafran

United States of America

Jianxin Bao

United States of America

Andrew Bateman

Canada

Christine Beattie

United States of America

Roland A Bender

Germany

Johannes Berger

Austria

Christophe Bernard

France

Gal Bitan

United States of America

Stephanie Booth

Canada

Scott T Brady

United States of America

Patrik Brundin

Sweden

Luigi Bubacco

Italy

Robert Burke

United States of America

Mark Burns

United States of America

Ashley Bush

United States of America

Ina Caesar

United States of America

Dongming Cai

United States of America

Eduardo Castano

Argentina

Anthony WS Chan

United States of America

Raymond Chuen-Chung Chang

Hong Kong

Sally Chappell

United Kingdom

Frederic Checler

France

Jeannie Chen

United States of America

John Cirrito

United States of America

Abbot Clark

United States of America

Casey Cook

United States of America

Mark Cookson

United States of America

Carlos Cruchaga

United States of America

Eva Czirr

United States of America

Luciano D'Adamio

United States of America

Pritam Das

United States of America

Ted Dawson

United States of America

Bart De Strooper

Belgium

Mariely DeJesus-Hernandez

United States of America

Paula Desplats

United States of America

Michael Deture

United States of America

Marc Diamond

United States of America

Chad Dickey

United States of America

Wenzhen Duan

United States of America

Martin Duennwald

United States of America

Nilufer Ertekin-Taner

United States of America

Steven Estus

United States of America

Jian Feng

United States of America

John Fryer

United States of America

Li Gan

United States of America

Tania Gendron

United States of America

Thomas Gillingwater

United Kingdom

Todd Golde

United States of America

Cheng-Xin Gong

United States of America

Gunnar Gouras

Sweden

Takuya Hayashi

Japan

Lawrence Hayward

United States of America

Niels Hellings

Belgium

Adel Helmy

United Kingdom

Peter Robin Hiesinger

United States of America

Hyang-Sook Hoe

United States of America

Elly M Hol

Netherlands

David Holtzman

United States of America

Yong Huang

United States of America

Nibaldo Inestrosa

Chile

Grazia Isaya

United States of America

Daisuke Ito

Japan

Kurt Jellinger

Austria

Hibiki Kawamata

United States of America

Rakez Kayed

United States of America

Myeong OK Kim

Korea, South

Jungsu Kim

United States of America

Anna King

Australia

Edward Koo

United States of America

Mary Jo LaDu

United States of America

Bruce Lamb

United States of America

Corinne Lasmezas

United States of America

Weidong Le

United States of America

Jiyeon Lee

United States of America

Jin-Moo Lee

United States of America

Malcolm Leissring

United States of America

Carla Lema Tome

United States of America

Cynthia Lemere

United States of America

Efrat Levy

United States of America

Yulong Li

United States of America

Yueming Li

United States of America

Xiao-Jiang Li

United States of America

Francesca-Fang Liao

United States of America

Charles Liberman

United States of America

Gérard Lizard

France

Seth Love

United Kingdom

Kurt Lucin

United States of America

David Lynch

United States of America

Stéphane Martin

France

Amelia Marutle

Sweden

María Paz Marzolo

Chile

Eliezer Masliah

United States of America

Pamela McLean

United States of America

Heather Melrose

United States of America

Makoto Michikawa

Japan

Carol Milligan

United States of America

Brit Mollenhauer

Germany

Kevin Morgan

United Kingdom

Sandra Mulder

Netherlands

Alysson Muotri

United States of America

Nobuki Nakanishi

United States of America

Robert Nickells

United States of America

Henrietta Nielsen

United States of America

Tomoyuki Nishizaki

Japan

Ralph A Nixon

United States of America

Anders Olsen

Denmark

Karen O'Malley

United States of America

Kalipada Pahan

United States of America

Francesc Palau

Spain

Udai Pandey

United States of America

Annalisa Pastore

United Kingdom

Kush Paul

United States of America

Robia Pautler

United States of America

Leonard Petrucelli

United States of America

Erik Portelius

Sweden

Domenico Pratico

United States of America

Marguerite Prior

United States of America

Lawrence Rajendran

Switzerland

Tomi Rantamäki

Finland

G William Rebeck

United States of America

Christiane Richter-Landsberg

Germany

Roberta Rigolio

Italy

Justin Rogers

United States of America

Terrone Rosenberry

United States of America

Owen Ross

United States of America

Kumar Sambamurti

United States of America

Wiep Scheper

Netherlands

Clemens Scherzer

United States of America

Yong Shen

United States of America

Jie Shen

United States of America

Mahendra Singh

India

Ioannis Sotiropoulos

Portugal

Nuno Sousa

Portugal

Nadia Stefanova

Austria

Jamuna Subramaniam

India

Keizo Sugaya

Japan

Tao Sun

United States of America

Leon Tai

United States of America

Haruo Takeshita

Japan

Davide Tampellini

Sweden

Zhiqun Tan

United States of America

Alexey Terskikh

United States of America

Giuseppina Tesco

United States of America

Roberto Testi

Italy

Dietmar Thal

Germany

Gopal Thinakaran

United States of America

Mathias Toft

Norway

Taisuke Tomita

Japan

Shichun Tu

United States of America

Brigita Urbanc

United States of America

Ludo Van den Bosch

Belgium

Klaus Van Leyen

United States of America

Robert Vassar

United States of America

Marcel Verbeek

Netherlands

Yoshinao Wada

Japan

Jochen Walter

Germany

Baiping Wang

United States of America

Xin Wang

United States of America

Edwin Weeber

United States of America

Andrew West

United States of America

Christian Wider

Switzerland

Micha Wilhelmus

Netherlands

Michael Willem

Germany

Juergen Winkler

Germany

Philip Wong

United States of America

Shaohua Xu

United States of America

Xuemin Xu

United States of America

Zuoshang Xu

United States of America

Shang-Hsun Yang

Taiwan

X William Yang

United States of America

Ikuru Yazawa

Japan

Midori Yenari

United States of America

Celina Zerbinatti

United States of America

Henrik Zetterberg

Sweden

Jianhua Zhang

United States of America

Yongjie Zhang

United States of America

Yun-wu Zhang

China

Zhuohua Zhang

China

Hui Zheng

United States of America

Xiongwei Zhu

United States of America

Haining Zhu

United States of America

